# Depression, anxiety, and stress in infertile couples during the COVID-19 pandemic: the consequences we face

**DOI:** 10.5935/1518-0557.20230018

**Published:** 2024

**Authors:** Anamarija Bogović, Ana-Meyra Potkonjak, Ivka Djaković, Hrvojka Soljačić Vraneš

**Affiliations:** 1 Department of Psychiatry, Sestre Milosrdnice University Hospital Center, Zagreb, Croatia; 2 Department of Psychology, Catholic University of Croatia, Zagreb, Croatia; 3 Department of Gynecology and Obstetrics, Sestre Milosrdnice University Hospital Center, Zagreb, Croatia; 4 Clinic for Gynecology, Bethesda Hospital, Basel, Switzerland

**Keywords:** depression, anxiety, stress, infertility, COVID-19

## Abstract

**Objective:**

Postponing assisted reproductive technology treatment can cause pronounced mental health problems. The aim of this study was to examine the level of depression, anxiety, stress, and overall infertility-related distress experienced by infertile couples during the pandemic, as well as the differences between men and women in the examined variables and the correlations between them.

**Methods:**

A total of 131 participants were included in the study, 65 men and 66 women. They were selected based on their responses in the Fertility Problems Inventory (FPI); the Depression, Anxiety, and Stress Scale-21 (DASS-21); and a general data questionnaire provided to them at the time of IVF.

**Results:**

The levels of depression, anxiety, and stress in women and men resided within the normal range. Depression (*p*<0.05), anxiety (*p*<0.01), stress (*p*<0.01), and social concern (*p*<0.05) were more pronounced among women. Significant correlations were found between depression, anxiety, stress, and global stress and its three dimensions: social concern, sexual concern, and relationship concern.

**Conclusions:**

During the pandemic, women undergoing assisted reproductive technology treatment experienced significantly higher levels of depression, anxiety, stress, and overall infertility-related stress than men. Furthermore, depression, anxiety, and stress were apparently correlated with overall infertility-related stress.

## INTRODUCTION

Infertility has been defined as “the inability to procreate, carry or deliver a baby naturally after having tried for at least one year” (Vander Borght & Wyns, 2018). An estimated 10-15% of the individuals of reproductive age have fertility problems (Vander Borght & Wyns, 2018).

Numerous studies found correlations between infertility and increased levels of stress, anxiety, and depression, as well as mental health issues in general (Rooney & Domar, 2018). However, the relationship between psychological distress and infertility is by no means simple. Although individuals with infertility may report higher levels of stress than those without fertility problems, it remains questionable whether psychological distress can lead to fertility difficulties (Greil, 1997). The literature shows that 25-60% of infertile individuals have psychiatric symptoms and higher levels of depression and anxiety than their fertile peers (De Berardis *et al*., 2014). Assisted reproductive technology (ART) treatment has also been associated with increased levels of depression and anxiety (García-Blanco *et al*., 2018), which tend to subside after treatment success (Matthiesen *et al*., 2011). Treatment postponement became more prevalent during the COVID-19 pandemic, along with mental health issues (Boivin *et al*., 2020).

The aim of the study was to examine the levels of depression, anxiety, stress, overall infertility-related stress in couples with fertility problems during the COVID-19 pandemic, as well as the differences between men and women in the examined variables and the correlations between observed variables.

## MATERIALS AND METHODS

This cross-sectional study included patients treated for infertility at the Sestre Milosrdnice University Hospital Center, a tertiary referral center, two months after the second major earthquake that hit Zagreb in 2020. In that specific time period, the clinic was temporarily inoperative due to the allocation of resources to deal with the substantial material damage caused by the earthquake and further complicated by the uncertainties of COVID-19. As the operations resumed, infertile couples were invited to answer three questionnaires: a general data questionnaire, the Depression Anxiety Stress Scale-21 (DASS-21), and the Fertility Problem Inventory (FPI). Statistical analysis was performed with the independent samples t-test and Pearson’s correlations. The local Institutional Ethics Committee approved the study.

### Instruments

#### Depression, Anxiety, and Stress Scale-21 (DASS-21)

The DASS-21 (Lovibond & Lovibond, 1995) is a shorter version of the original scale, which contained 42 items. This scale consists of 21 items and includes three subscales, each with seven items: depression (e.g*. “I felt I had nothing to look forward to”*), anxiety (e.g. “*I felt I was close to panic”*), and stress (e.g. “*I found it hard to wind down”)*. Participants are asked to estimate how much each particular statement referred to them over the past week and rate them on a 4-point Likert scale (0 - did not apply to me at all, 1 - applied to me to some degree, or some of the time, 2 - applied to me a considerable degree or a good part of the time, 3 - applied to me very much or most of the time). The result is the summation of the points multiplied by two, giving a result between 0 and 42. Higher results indicate a higher level of depression, anxiety, and stress. This scale possesses great reliability of internal consistency. The calculated Cronbach’s alpha values in a study by Crawford & Henry (2003) were α=0.90 for the anxiety subscale, α=0.95 for the depression subscale, α=0.93 for the stress subscale, and α=0.97 for the overall scale. Cronbach’s alpha values in this study were α=0.88 for the depression subscale, α=0.85 for the anxiety subscale, and α=0.92 for the stress subscale.

#### Fertility Problem Inventory (FPI)

The FPI *(Newton et al., 1999) measures perceived infertility-related stress in five dimensions: social concern, sexual concern, relationship concern, rejection of childfree lifestyle, and need for parenthood. The total score portrays global stress related to infertility. The Inventory consists of 46 items scored on a 6-point scale (1 - completely disagree, 6 - completely agree). Some items are reversely coded. Higher scores indicate higher levels of stress.* Cronbach’s alpha values on a Croatian population were 0.79, 0.83, 0.78, 0.80, and 0.85 for social concern, sexual concern, relationship concern, rejection of childfree lifestyle and need for parenthood, respectively. The Cronbach’s alpha for the whole scale was 0.90 (Nakić Radoš *et al*., 2022).

#### General data questionnaire

Sociodemographic information about age, marital status, education, occupation, property and income, area of residence, and earthquake exposure were collected in the general data questionnaire.

## RESULTS

### Participants

A total of 131 participants - 65 men and 66 women - with fertility problems were included in the study. Participant mean age was 35.115.06 years. Almost three quarters (74%) were married and 26% lived in extramarital unions. On average, the couples had been together for 9.3 years and had been married/living together for 5.4 years. Most of them (55.7%) had a high school degree, 42% had college degrees, and 2.3% finished primary school. Most were employed (89.3%). Household income was rated as average by 73.3% of the participants, as slightly below average by 1.5%, significantly below average by 0.8%, as slightly above average by 21.4%, and significantly above average by 2.3% of them. More than half (50.4%) of the participants live in a city with more than 100,000 people, 22.4% in a city with up to 100,000 people, and 26.2% in the countryside. Regarding the cause of infertility, 32.1% reported female factors, 17.6% reported both female and male factors, 13.7% reported male factors, and 36.6% reported unknown factors. The majority had been treated with in vitro fertilization (IVF) (48.1%), 23.7% with insemination, 8.4% with IVF and insemination, 1.5% with ICSI, and 17.6% had not been treated yet. Almost two thirds (64.1%) experienced the earthquake, while 35.9% did not.

### Depression, anxiety, and stress in infertile couples and infertility-related stress

Mean values and standard deviations of the observed variables are shown in Table 1, as well as the *p*-values reported from an independent samples t-test (differences between male and female participants).

**Table 1:** Means, standard deviations, and *p*-values from t-test.

	All participants Mean; SD	Female participants Mean; SD	Male participants Mean; SD	*p*-value
Depression	5.08: 6.8	6.3; 7.3	3.85; 6.1	.038^*^
Anxiety	3.05; 4.6	4.21; 5.4	1.88; 3.4	.003^**^
Stress	8.61; 7.7	10.55; 8.4	6.65; 6.3	.003^**^
Social concern	20.55; 8.5	22.06; 8.4	19.03; 8.3	.042^*^
Sexual concern	14.91; 8.2	16.06; 8.4	13.77; 7.9	.112
Relationship concern	18.97; 7.9	19.7; 7.9	18.25; 7.9	.300
Rejection of childfree lifestyle	30.34; 9.1	30.45; 8.7	30.23; 9.6	.894
Need for parenthood	36.59; 9.9	37.02; 10.2	36.16; 9.6	.621
Global stress	119.68; 28.3	122.77; 29.4	116.84; 27.11	.256

* significant at the 0.05 level

** significant at the 0.01 level.

The recommended cutoff scores for conventional severity labels indicated that study participants had normal depression, anxiety, and stress levels (Lovibond & Lovibond, 1995).

*An independent samples t-test* was performed to examine differences in depression, anxiety, and stress levels, social concern, sexual concern, relationship concern, rejection of childfree lifestyle, need for parenthood, and global stress between male and female participants.

Results showed a statistically significant higher level of depression, anxiety, stress, and social concern in female participants.

Pearson’s correlation coefficients were calculated for depression, anxiety, stress, global stress, and five dimensions of perceived infertility-related stress (social concern, sexual concern, relationship concern, rejection of childfree lifestyle, and need for parenting). Results are in Table 2.

**Table 2:** Pearson’s correlation coefficients between depression, anxiety, stress, global stress, and five dimensions of infertility-related perceived stress (social concern, sexual concern, relationship concern, rejection of childfree lifestyle, and need for parenting).

	1.	2.	3.	4.	5.	6.	7.	8.	9.
1. Depression		.665^**^	.814^**^	.501^**^	.510^**^	.435^**^	.111	.139	.441^**^
2. Anxiety			.680^**^	.496^**^	.336^**^	.263^**^	.038	-.042	.300^*^
3. Stress				.497^**^	.436^**^	.374^**^	.103	.080	.386^**^
4. Social concern					.553^**^	.379^**^	.125	.131	.619^**^
5. Sexual concern						.679^**^	.165	.238^**^	.743^**^
6. Relationship concern							.163	.244^**^	.677^**^
7. Rejection of childfree lifestyle								.550^**^	.623^**^
8. Need for parenthood									.668^**^
9. Global stress									

** correlation is significant at the 0.01 level (2-tailed)

* correlation is significant at the 0.05 level (2-tailed).

There were statistically significant correlations between depression and anxiety, stress, global stress, and three dimensions of infertility-related perceived stress: social concern, sexual concern, and relationship concern. Anxiety was significantly correlated with stress, global stress, social concern, sexual concern, and relationship concern. Statistically significant correlations were also found between stress and global stress, social concern, sexual concern, and relationship concern. Global stress was significantly correlated with all dimensions of infertility-related perceived stress; social concern was significantly correlated with sexual and relationship concern; sexual concern was significantly correlated with relationship concern; relationship concern was significantly correlated with rejection of childfree lifestyle; rejection of childfree lifestyle was significantly correlated with need for parenthood.

## DISCUSSION

Differently from previous studies, all participants had normal levels of depression, anxiety, and stress (Volgsten *et al*., 2008; Sejbaek *et al*., 2013; Pasch *et al*., 2016).

Levels of anxiety, depression, and stress were significantly higher in women. This might be due to the ability women have to express emotional difficulties related to mental health more easily than men. Most studies found that depression was twice as common in women than in men (Cyranowski *et al*., 2000). In females, this phenomenon was partially explained by the impact of gonadal hormones on depression, especially in the postpartum period and menopause.

In terms of gender differences of patients being treated for infertility, higher levels of depression, cognitive dysfunction, and anxiety were found in women compared with men. Conversely, men experienced psychosomatic distress and stress more often, which correlate with lower quality of life. However, men had higher scores in relationship satisfaction (Simionescu *et al*., 2021). Newton *et al*. (1999) found significantly higher values on scales of global stress, social concern, sexual concern, and need for parenthood in female patients with infertility than in male patients. This is in line with our findings, in which female participants had higher scores on all measured variables, although women only differed significantly on the social concern dimension compared to men. A large body of previous studies also found similar results (Chehreh *et al*., 2019; Patel *et al*., 2016). Our results showed a statistically significant higher level of depression, anxiety, stress, and social concern in female participants. However, literature reports indicate that men are seeking fertility treatment as often as women and experiencing high levels of depression (Holley *et al*., 2015). Furthermore, women were found to experience higher stress levels during infertility treatment than men. They experience higher levels of depression and anxiety, general psychological problems, and lower self-esteem compared with men (El Kissi *et al*., 2013). In our study, all patients experienced normal levels of depression, anxiety, and stress.

The question is why the depression, anxiety, and stress levels in our population were rated as normal, since previous studies indicated and the pandemic and the earthquake were correlated with increases in mental health issues (Peitl *et al*., 2020; Thapa *et al*., 2018). Studies examining groups of patients waiting for non-urgent gynecological surgery during the time of the pandemic and the earthquake found normal levels of depression, anxiety, and stress, which were attributed mainly to higher quality of life (Soljačić Vraneš *et al*., 2022). A possible explanation for the low levels of depression, anxiety, and stress in this population is the hope associated with the initiation of assisted reproduction treatment. Additionally, self-report measures have their limitations and may not be entirely accurate.

Infertility-related perceived stress in all its dimensions - social concern, sexual concern, relationships concern, rejection of childfree lifestyle, and need for parenthood - were significantly correlated with depression. Anxiety was significantly correlated with stress, global stress, and three dimensions of infertility-related perceived stress (social concern, sexual concern and relationship concern). These results were consistent with previous research (Wang *et al*., 2015). Moura-Ramos *et al*. (2012) also found positive correlations between depression, anxiety, and all dimensions of the Fertility Problem Inventory. In other words, higher scores on depression and anxiety scales were correlated with higher scores on measures of infertility-related perceived stress and their dimensions. Our results were consistent with the findings of Newton *et al*. (1999). Our results, as well as the studies mentioned above, confirmed the idea that infertility is a stressful experience that can cause mental health issues.

The limitations of the study include the fact that the questionnaires were answered by couples attending the clinic regardless of treatment stage (first appointment, scheduled ultrasound examination, time after oocyte retrieval, etc...). Each of these events may trigger acute reactions. Nevertheless, our results may facilitate the understanding of the emotional issues faced by infertile couples, especially in challenging times such as the pandemic and the earthquake.
